# Magnetic resonance imaging during life: the key to unlock cerebral malaria pathogenesis?

**DOI:** 10.1186/1475-2875-13-276

**Published:** 2014-07-18

**Authors:** Sanjib Mohanty, Terrie E Taylor, Sam Kampondeni, Mike J Potchen, Premanand Panda, Megharay Majhi, Saroj K Mishra, Samuel C Wassmer

**Affiliations:** 1 Ispat General Hospital, Rourkela 769005, Orissa, India; 2 Department of Osteopathic Medical Specialties, College of Osteopathic Medicine, Michigan State University, East Lansing, MI 48824, USA; 3 Blantyre Malaria Project, University of Malaŵi College of Medicine, Blantyre 3, Malaŵi; 4 Department of Radiology, Queen Elizabeth Central Hospital, Blantyre, Malaŵi; 5 Department of Imaging Sciences, University of Rochester Medical Center, Rochester, NY 14642, USA; 6 Department of Microbiology, Division of Parasitology, New York University School of Medicine, 341 East 25th Street, New York NY 10010, USA; 7 Department of Pathology K25, Sydney Medical School, The University of Sydney, Sydney NSW 2402, Australia

**Keywords:** *Plasmodium falciparum*, Cerebral malaria, Pathophysiology, Neuroimaging, MRI

## Abstract

Understanding the mechanisms underlying the pathophysiology of cerebral malaria in patients with *Plasmodium falciparum* infection is necessary to implement new curative interventions. While autopsy-based studies shed some light on several pathological events that are believed to be crucial in the development of this neurologic syndrome, their investigative potential is limited and has not allowed the identification of causes of death in patients who succumb to it. This can only be achieved by comparing features between patients who die from cerebral malaria and those who survive. In this review, several alternative approaches recently developed to facilitate the comparison of specific parameters between fatal, non-fatal cerebral malaria and uncomplicated malaria patients are described, as well as their limitations. The emergence of neuroimaging as a revolutionary tool in identifying critical structural and functional modifications of the brain during cerebral malaria is discussed and highly promising areas of clinical research using magnetic resonance imaging are highlighted.

## Background

Globally, an estimated 3.3 billion people were at risk of malaria in 2011, with populations living in sub-Saharan Africa representing approximately 80% of cases and 90% of malaria-related deaths [1]. *Plasmodium falciparum* is a major cause of morbidity and mortality worldwide. Neurological complications are common in falciparum malaria, but cerebral malaria (CM) is the most severe and is associated with almost all neurocognitive sequelae. This complex and multi-factorial syndrome is characterized by a potentially reversible encephalopathy with coma and leads to a mortality rate of approximately 15%-25% even when appropriate treatment and intensive care are provided. The fundamental pathogenesis of fatal CM is still not well understood. However, several hypotheses have been advanced, including impaired tissue perfusion due to mechanical obstruction of microvessels by *P. falciparum*-parasitized red blood cells, hyper-activation of host immune cells leading to the excessive release of potentially harmful pro-inflammatory cytokines [2-4], as well as coagulation dysfunction [5,6]. These hypotheses may explain of some aspects of disease pathogenesis, but they fail to explain the extent and reversibility of the coma associated with CM. Due to the lack of specific neuro- or vasculoprotective therapies, treatments are currently still limited to anti-malarial drugs and emergency supportive care [7]. Elucidation of key processes may suggest avenues for new interventions that will improve the ability of clinicians to recognize CM and decrease the mortality rate. The use of animal models is helpful in exploring new hypotheses, but is limited as most of the models do not approximate the disease closely enough to allow extrapolations [8].

In this review, the limitations of approaches developed recently to circumvent the problems of traditional pathology analyses are briefly described and the different neuroimaging techniques used to date to investigate the pathogenic mechanisms associated with the development of CM are compiled, with a particular focus on the promising results obtained with magnetic resonance imaging (MRI). In addition, pivotal areas of future research are highlighted, with the aim of implementing new evidence-based therapeutic avenues and improving outcomes.

### The long road to neuro-imaging

#### Post-mortem: the classical approach and its limitations

Historically, basic information regarding the pathogenesis of CM has been generated by integrating autopsy findings with clinical information gathered during life. For example, Alphonse Laveran, who discovered the malaria parasite [9], observed that the organ-specificity of the cerebral syndrome corresponded to the burden of malaria parasites accumulated in the brain [10]. Detailed quantitative histological studies later supported this relationship [11]. Marchiafava and Bignami deduced that parasite-infected erythrocytes (IE) bind to endothelial surfaces, leading to the reported sequestration [12]. Such clinico-histopathology studies – some of them are still ongoing – have led to the discovery of several pathological features that are believed to be crucial in the development of CM [13-16].

However, the autopsy approach has major limitations. With few exceptions, which include the Malaŵi series of 103 autopsies [14], the observations reported during post-mortem studies in the field were obtained from a small number of CM patients. This is largely due to the colossal logistical and financial challenges involved in maintaining a well-coordinated, long-term and multi-disciplinary capacity to characterize patients clinically prior to death, perform comprehensive autopsies, and analyse the material collected during the post-mortems. Second, cultural and/or religious taboos may increase the reluctance of families to authorize autopsies, making it difficult to perform post-mortem analyses on large-scale in some endemic areas. Indeed, while some of the pioneering work in CM pathology was performed in South East Asia [17-21], there is currently no extensive quantitative clinico-histopathology study of adult CM ongoing in this region. The largest reported repository of autopsy samples in South East Asia was collected in Vietnam during the mid-1990s [22-24], before the presence of retinopathy was recognized as a crucial component of the accurate diagnosis of both paediatric and adult CM [25-27]. Thus, the extensive comparison of pathological findings between carefully clinically defined African and Asian patients who died of CM has not been possible so far.

Lastly, two inherent limitations of autopsy-based studies of malaria now restrict our capacity to move beyond describing associations: i) data are generated from a single point in time, precluding the identification of relevant pathogenic processes; and ii) tissue is only available from individuals who die, preventing any comparisons with survivors. Indeed, without being able to compare those who survive to those who die it is not possible to know if the observed pathology in patients dying of CM is sufficient to cause death.

#### Alternative approaches: getting closer to the brain

In order to evade the limitations discussed above, tremendous efforts have been made over the past decade to develop novel and non- or minimally-invasive approaches, allowing for the first time the comparison of specific features between fatal CM and uncomplicated malaria patients. For example, *ex vivo* techniques were recently used to evaluate potential differences in endothelial predisposition to activation by tumour necrosis factor in paediatric patients from Malaŵi. Because endothelium of the adipose tissue closely resembles cerebral endothelium [28], it represents a useful *ex-vivo* model for examining brain endothelial dysfunction in the context of CM [29]. Endothelial cells were, therefore, obtained from subcutaneous fat samples for analyses. This was efficiently performed by needle aspiration biopsy on Malaŵian patients with CM or uncomplicated malaria, a fast, simple and painless procedure after the application of local anaesthetic [29]. Although the use of this model proved successful within the parameters of the study, it presents obvious restrictions and the lack of tight junctions in the vasculature of the adipose tissue does not allow the in-depth investigation of pathophysiological mechanisms believed to play a key role in fatal paediatric CM. These include endothelial injury, blood–brain barrier dismantlement and intracranial hypertension [30,31].

Another example of an alternative advance is the use of orthogonal polarization spectral (OPS) imaging to assess the microcirculatory changes in mucosal surfaces during malaria infection [32]. The clear images of sublingual and rectal mucosa generated by the OPS device allowed for the first time the visualization *in vivo* of microvessel obstruction by IE in adult patients with severe malaria, confirming the evidence derived from pathological studies of fatal cases [33]. While this model offers the advantage of a real time intravital analysis of peripheral sequestration processes that can be easily applied to both CM and uncomplicated malaria patients, it also presents limitations. Indeed, the profile of expressed surface receptors is a key determinant of endothelial function and this phenotype differs markedly between organs [34]. The sequestration observed in the mucosa of infected patients may therefore not reflect accurately what happens in the cerebral microvessels, as those two vascular beds express different receptors for IE.

This organ-specific endothelial phenotype pitfall was elegantly circumvented by the examination of the retina in malaria patients, a recent approach undertaken as part of the redefinition of CM syndromes to increase the accuracy of its diagnosis [35]. The retina is embryologically related to the central nervous system and its microvasculature displays an analogous cellular structure and blood-tissue barrier. Its examination, therefore, provides a unique opportunity to study the microvasculature of infected patients and the effects of IE sequestration on neurologic tissue *in vivo*. Ocular abnormalities, now known as malarial retinopathy, were described in patients with CM, both in African children [36-39] and Bangladeshi adults [40,41]. When compared with less severe malaria patients, the prevalence and intensity of malarial retinopathy was proportional to the intensity of the clinical syndrome [26,42]. It has three main components: retinal whitening, vessel changes and retinal haemorrhages. Retinal whitening closely resembles the patchy ischemic retinal changes in central vein occlusion [43] and matches areas of hypo-perfusion, likely related to the presence of IE sequestrated in the retinal microvasculature [44]. This approach has proved critical in the accurate diagnosis of CM [25,35] and although the presence of any one of the three features of malaria retinopathy is sufficient to make the diagnosis, retinal whitening is at present the most sensitive and specific clinical indicator of cerebral sequestration [45]. In addition, a recent analysis of the optic nerve sheath by ultrasound allowed the measure of increased intracranial pressure in Malaŵian children with CM [46], a common feature of the syndrome. Despite the knowledge leap afforded by retina examination during life, the procedure presents investigative restrictions and has not allowed the identification of the cause of death in CM patients who succumbed. Indeed, the clinical findings gathered through the eye examination to date do not reflect the occurrence of potentially lethal cerebral events associated with CM such as axonal injury [47], nor do they allow the prediction of acute mortality in comatose patients [48].

#### Neuroimaging during life: the answer to all the questions?

CM occurs predominantly in resource-poor settings, where advanced medical imaging technologies are generally unavailable. The emergence of neuroimaging as a tool in elucidating the pathophysiology of the neurologic syndrome has therefore been a long and tortuous process. After being restricted to computed tomography (CT) technology for a long time, neuroimaging studies recently started to use MRI approaches on a large scale to enhance the clinical characterization of the disease. Globally, the use of more sophisticated investigative and non-invasive medical imaging technologies could transform our understanding of pathophysiological derangements in critical illness and result in substantial improvements in acute management of patients with CM.

#### Computed tomography: a first step

CT scans are now commonly available, do not require a challenging infrastructure, are cheap to run and allow a snapshot of the patient’s brain in less than a minute. In addition, contrast material can be administered to increase conspicuity of vascular lesions and breakdown of the blood brain barrier. The first report of the use of CT in malaria patients was published in 1983 and scans of the brain in 10 Thai adult patients with CM revealed mixed results. Evidence of cerebral oedema was only detected during the agonal phases in two adults, while four patients had completely normal scans. The authors concluded that oedema might occur in CM but cannot always explain the occurrence of coma in patients [49]. After a hiatus of over a decade, a similar study was performed in 14 African children with CM. CT was carried out both during the recovery phase and at follow-up and similar discrepancies were observed, albeit at a higher ratio. Six children had diffuse brain swelling and eight patients exhibited normal scans [50]. These differences might be explained by the rapid resolution of brain swelling in children, coupled to the late monitoring of CT changes. As part of a subsequent study in India, 21 patients diagnosed with CM underwent both non-enhanced and contrast CT. The occurrence of diffuse brain swelling was observed in 42% of the patients and was associated with a poor prognosis, whereas no deaths were recorded in patients with normal CT findings [51]. Finally, the recent CT monitoring of a larger cohort of 126 Indian patients with CM revealed that brain swelling is the most common finding associated with the pathology. Indeed, 63% of the patients had cerebral swelling, the amplitude of which was not, however, related to coma depth or mortality [52].

CT scans have certainly illuminated some of the pathological changes associated with CM in both adult and paediatric patients but there is no unanimity regarding the occurrence of brain swelling or its role in the induction of coma. Such discrepancies could be due to the overall small number of patients enrolled, the lack of controlled studies, the different points at which CT scans have been performed or the absence of contrast enhancement in the largest series. In addition, none of these studies incorporated the presence of retinopathy as a diagnostic tool for CM and might, therefore, have included a substantial proportion of patients with non-malarial central nervous system disorders [25]. Indeed, a study performed in Malaŵi and restricted to retinopathy-positive CM patients demonstrated that acute CT scans reveal findings consistent with autopsy reports, and that the presence of diffuse brain swelling, whether it involves the brainstem or not, was associated with protracted coma and death [53].

**Figure 1:**
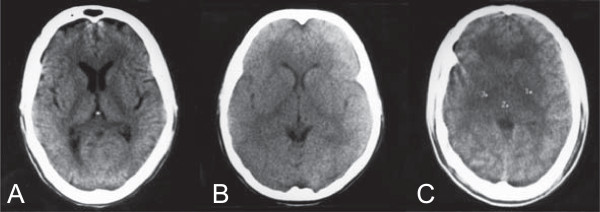
**CT scans of adult CM patients from India showing mild to severe brain swelling.** Non-enhanced 8 mm transverse sections of a control patient **(A)** and two acute CM patients showing mild and severe cerebral oedema (**B** and **C**, respectively). Images were obtained within 6 hours of admission at Ispat General Hospital in Rourkela, Orissa, India, as part of another study [52] and using a Philips W1000 third generation CT scan. Brain swelling observed in **(B)** and **(C)** was defined as loss of cerebrospinal fluid space, small ventricles, absence of sulci, and/or compression of the cisterns. Mild brain swelling was defined as local or diffuse effacement of sulci and Sylvian fissure without compression of the supratentorial ventricular system **(B)**. Severe swelling was defined as diffuse obliteration of sulci, Sylvian fissure and basal cisterns, together with an important mass effect causing near total obliteration of the supratentorial ventricular system and generalized hypodensity of brain parenchyma **(C)**.

The most consistent finding in all the studies is the presence of brain swelling in some CM patients (Figure 1). This could be attributed to the increased blood volume in the brain following the sequestration of IE, but also to the accumulation of intra-cellular fluid due to impaired cellular metabolism (cytotoxic oedema) and the accretion of interstitial fluid in brain tissue following a breakdown of the blood–brain barrier (vasogenic oedema). It is likely that these mechanisms are linked, since cytoadherence of IE to endothelial cells has been shown to compromise the functional integrity of the blood–brain barrier [54]. The most significant limitation of CT technology for the study of CM pathophysiology is the inability to distinguish vasogenic from cytotoxic oedema. This distinction can, however, be made by diffusion-weighted MRI scanning and is crucial to shed light on the possible mechanisms that may be leading to brain swelling in CM, as well as evaluate new therapeutic avenues.

#### Magnetic resonance imaging – the finish line?

MRI is a powerful diagnostic tool for monitoring and understanding key features involved in the progression of neurologic diseases. It was, therefore, identified as the most promising imaging modality for visualization of CM pathology *in vivo*[55,56]. However, this approach has been mainly restricted to animal models of CM and until recently, almost all clinical MRI studies have involved a single case report of small series of patients [57-62]. These reports presented variable results, which can be attributed to the low number of patients involved in the studies, the non-specific neurological manifestations generated by the diffuse involvement of the brain, the variation in patient age and the evolution of MRI techniques and resolution over the years.

**Figure 2:**
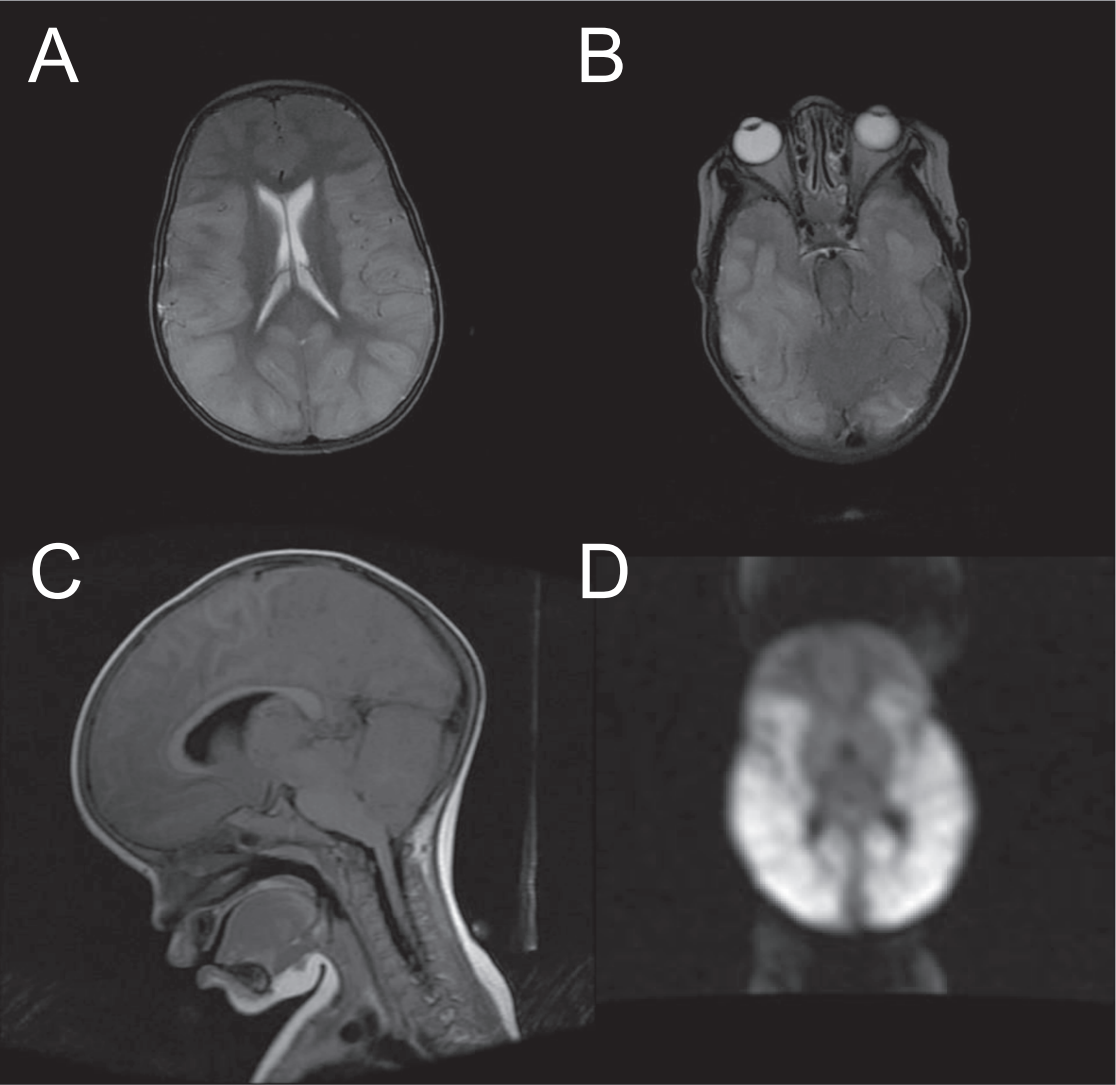
**MRI of paediatric patient with fatal CM from Malaŵi presenting severe brain swelling.** Admission axial T2 scan (TR 3140, TE 123) of a 4 years old male child with fatal retinopathy-positive CM performed at Queen Elizabeth Central Hospital in Blantyre, Malaŵi **(A)**. Severe brain swelling is observed, with complete loss of sulcal markings. **(B)** shows a lower slice depicting severe brainstem compression resulting from the swelling. A mid-sagittal T1 Flair image (TR 2311, TE 26, ET 2) of the same patient reveals the complete effacement of all basilar cisterns and the 4^th^ ventricle, as well as severe brainstem compression **(C)**. Finally, **(D)** shows potential diffuse restricted diffusion in the cerebral cortex and white matter. The child succumbed from respiratory failure 3 hours after these images were obtained.

The first MRI study of a large series of patients with malaria living in an endemic area was performed in Thailand in 1995 in adults using a 0.2 Tesla scanner. It revealed that increased brain volume and swelling was common in patients with CM, but this difference was attributed to an increase of the volume of intracerebral blood caused by sequestration of IE rather than cerebral oedema [63]. MRI techniques have developed substantially since the publication of these results and clinical MRI technology is now available in several endemic countries. MRI in resource-limited settings is challenging due to specific infrastructure requirements including sufficient foundation to withstand the heavy magnet, continuous power supply, steady climate control, access to helium, and digital image archiving capabilities [64]. Despite these challenges, several MRI centers have been successfully installed and can be used to enhance the clinical characterization of patients with CM through application of neurological MRI methods. A milestone in this approach was reached in June 2008, when a 0.35 Tesla MRI unit became available in Malaŵi, leading to the largest prospective study of paediatric CM ever undertaken to date [65]. Indeed, the use of a low-field permanent magnet MRI is a solution to some of the hindrances stated above, in particular frequent power outages and the unpredictable availability of cryogens could result in the quenching of a high field superconducting magnet. Initially, 152 children with acute CM were enrolled and 120 children meeting a stringent retinopathy-positive definition of CM were compared to 32 retinopathy-negative CM patients. A wide range of structural abnormalities was identified in the brain of the retinopathy-positive group, which is consistent with the high variability of findings reported at autopsy [14]. The significant findings included basal ganglia changes, white matter changes, pons and brainstem changes in addition to diffuse cortical involvement. The disease affected the posterior fossa less frequently. This descriptive study did not focus on clinical correlates beyond retinopathy status, but a moderate to severe increase in brain volume was identified as the single most important discriminator between retinopathy-positive and retinopathy-negative patients (Figure 2). Interesting and unusual patterns of restricted diffusion were reported in the retinopathy-positive group in various sub-segments of the brain [65].

**Figure 3:**
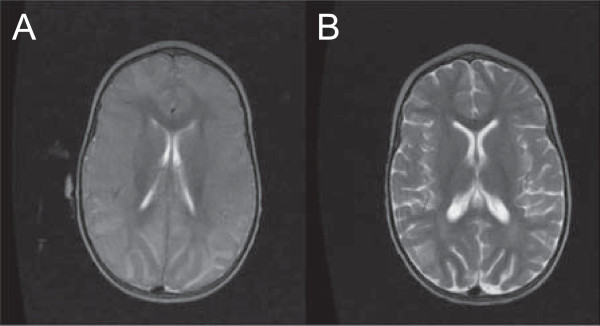
**MRI of paediatric CM survivor from Malaŵi during the acute phase and at follow-up.** Admission axial T2 scan (TR2960, TE122) of a 2 years old male Malaŵian child with retinopathy-positive CM performed at Queen Elizabeth Central Hospital in Blantyre, Malaŵi. There is severe brain swelling with total loss of sulcal markings and grey-white differentiation **(A)**. The same child presents mild diffuse atrophy one month later during the post-discharge follow-up visit **(B)**.

This first study had several limiting factors, including the absence of serial analyses, which is presently underway, and an unexpected inability to detect contrast agent. Since MRI routinely shows contrast enhancement at low fields, the lack of such a finding may be related to the status of the blood-brain barrier during paediatric CM. Further research into the integrity of this permeability barrier as a source for non-enhancement is presently being undertaken using contrast in paediatric CM patients on the high field MRI located in neighboring Zambia. Further research into the integrity of this permeability barrier as a source for non-enhancement is presently being undertaken using contrast in paediatric CM patients on the high field MRI located in neighbouring Zambia. However, the results generated are in line with previous reports in adult CM MRIs [58] and CT scans in both paediatric [50] and adult patients [51,52], suggesting that increased brain volume may be a common feature in CM, and that it may be implicated in the pathogenesis of death in patients with CM. This could also explain its reversibility, as brain swelling decreases significantly during recovery (Figure 3). The underlying mechanism for this increased brain volume remains unclear and large-scale analyses including serial imaging and evaluation of diffusion-weighted imaging findings, as well as clinical, retinopathy and electro-encephalographic data collection during the acute coma phase and at both short and long term follow-up are ongoing and aimed at addressing these questions.

Interestingly, a high-field (3.0 Tesla) MRI series of acute uncomplicated falciparum malaria patients from Thailand recently reported that four out of 10 patients presented a lesion with restricted water diffusion in the splenium of the corpus callosum [66]. These lesions were transient and similar in signal and location to some described in CM patients from the Malaŵi study [65], showing for the first time that acute cerebral injury does also occur in the absence of the neurologic syndrome. Using diffusion-weighted imaging to compare the intensity of these lesions between acute uncomplicated patients and CM patients both in larger paediatric and adult populations may, therefore, represent a major step in elucidating mechanisms underlying the pathophysiology of CM.

While the systematic application of MRI techniques to CM patients represents a revolutionary new approach, there are predictable limitations. For instance, it is entirely possible that factors inherent in the clinical presentation of the patients directly or indirectly effect quantitative MRI imaging and confound the investigation of CM pathogenesis. The multiple organ failure commonly observed in adult patients with CM in Asia is a complication that may lead to systematic changes, possibly resulting in MRI signatures not associated with the occurrence of the cerebral syndrome. Discriminating between “pure” CM patients, with no associated complications, and “broad” CM patients, with one or more associated complications, may help circumvent this potential bias. In addition, the MRI scan length also presents potential clinical management issues, especially when the full sequence includes perfusion and spectroscopy analyses. While this is unlikely to be problematic in stable comatose patients, it might prove challenging in closely monitored patients, as well as pre-comatose ones. Finally, the use of gadolinium-based contrast agents needs to be assessed rigorously. For safety issues, it cannot be used in patients with acute renal failure, a common complication of severe *P. falciparum* infection in adults. Alternative contrast agents have recently become available and could allow the MRI analysis of such patients in the future. All of these limiting factors need to be carefully considered for any prospective study of CM patients using MRI techniques.

## Conclusions

Magnetic resonance examination of both adult and paediatric patients infected with *P. falciparum* could represent a major milestone in the study of CM pathology. Until now, a comparison of both age groups has not been performed, despite the distinct differences in terms of clinical course, pathophysiology and autopsy findings [67]. In terms of MRI features, the amount of available data is currently fractional in adult CM due to the small number of reported cases. A comparison between the Malaŵi series and these reports would, therefore, be uninformative, owing to i) the lack of protocol and sequence standardization between the different sites*, ii)* the great disparity in patient numbers and iii) the subacute nature of the MRI studies in adults.

In order to circumvent these discrepancies, new collaborative efforts between MRI-equipped sites located in Asia and in Africa are required, with standardized clinical protocols and using the presence of retinopathy as a diagnostic parameter in CM patients. This will allow not only the clinical characterization of both paediatric and adult CM through the application of neurological MRI methods, but also a precise comparison between carefully clinically defined cohorts of patients from different age groups and from different continents.

While the magnet used in the Malaŵi series does not permit the visualization of ring haemorrhages, more modern systems with higher field strength magnets are now available in several clinical sites in Asia, allowing increased resolution, thinner slice imaging, and diffusion imaging, which should show ischemia, diffusion tensor imaging to show axons via tractography and damaged white matter via fractional anisotropy maps, spectroscopy to follow lactate in ischemia, susceptibility weighted imaging which may reveal haemozoin, and perfusion imaging which may demonstrate occluded arterioles. Potential pitfalls due to differences in magnetic field of MRI scanners between sites can be avoided by using common specific sequences. In addition, in order to ensure the accurate interpretation and comparison of MRI findings in these sequences, all the images can be scored and shared between radiologists via web-based platforms to ensure the highest diagnosis accuracy [68].

Such extensive MRI techniques have never been applied systematically to patients with acute complicated or uncomplicated malaria and present a highly promising approach to further investigate the causal relationship between brain swelling and the onset of CM. This can be achieved by assessing MRI patterns supporting or refuting the potential contribution of key pathogenic mechanisms of cerebral oedema in patients with CM. Finally, comparison studies between fatal cases of CM and survivors will, hopefully, lead to the identification of causes of death.

## Competing interests

All authors declare they have no competing interests.

## Authors’ contribution

SM, TET, SK and SCW searched the relevant literature. SM and SCW wrote the first draft of the manuscript. All authors appraised and revised the manuscript. All authors gave final approval for submission of the manuscript.
